# More Convoluted Than a Brazilian Soap Opera: How an Eager Chemistry Professor and a Well-Intended but Misguided Federal Judge Ignited an Industry of False Hopes

**DOI:** 10.1200/JGO.2015.002998

**Published:** 2016-02-10

**Authors:** Gustavo dos Santos Fernandes, Gilberto de Lima Lopes

**Affiliations:** **Gustavo dos Santos Fernandes,** Brazilian Society of Clinical Oncology and Oncology Center Hospital Sírio-Libanês Brasília, Belo Horizonte, Brazil; and; **Gilberto de Lima Lopes Jr,** Oncoclinicas do Brasil Group, São Paulo, Brazil, and Johns Hopkins University, Baltimore, MD.

If you pry into a Brazilian household around 10 pm on a weeknight, odds are that at least one television set will be tuned in to Globo TV, a broadcasting behemoth whose programs are watched by 90 million people every day. Its chief attractions are soap operas. Ranging in themes from historic productions to more modern issues, such as living through an economic downturn, these usually run over a period of a few months and have multiple characters and concomitant stories. But not even the most creative of soap opera authors could have thought of a story as convoluted as the one that has been unfolding in the last semester of 2015 and that oncologists have come to call phosphogate.

ANVISA, the health surveillance agency of Brazil, has a strict regulatory process for the registration of new drugs, which is often criticized for being too time consuming and rigorous. When it comes to cancer drugs, the agency has historically given marketing approval only to those medications that have been assessed in powered randomized clinical trials and does not accept surrogate end points other than overall survival. As such, medications available worldwide, such as lenalidomide and afatinib, are not considered effective enough and are unavailable to Brazilians. Regulatory issues also plague the conduct of clinical trials because investigators and sponsors are required to submit protocols not only to institutional ethics committees but also to a national one, CONEP (Comissão Nacional de Ética em Pesquisa). This dual process generates delays, with a lead time of up to 12 months before a trial can begin enrollment. These strict regulations are currently being challenged for access to an unproven substance that is reputed to have antineoplastic activity: synthetic phosphoethanolamine.

A key precursor of the biosynthesis of phospholipids in the cellular membrane, and part of the cell signaling system either directly or via second messengers, phosphoethanolamine, in its synthetic version, has been studied by a chemistry professor in one of the campuses of the University of São Paulo, one of the most prestigious universities in Brazil. Potential antineoplastic effects have been demonstrated in a few preclinical studies^1-4^ in cell lines and mice models^5^; nonetheless, no appropriate safety studies have been carried out in animals or humans. That has not kept the eager professor from manufacturing the substance in one of the chemistry laboratories of the university (which is not registered for producing medicines) and distributing it to an increasing number of patients over the years; he claims that nearly 40,000 individuals have been treated (Table 1). In September 2015, after he retired, the university administration finally decided to stop this nonsensical practice. What appeared to be the end of a problem that did not even merit a footnote in history books actually sparked a spate of lawsuits, forcing the university to maintain the production and distribution of the substance not only to patients who were already using it but also to new ones who were motivated by the attention generated by the news media when production was suspended.

**Table 1:**
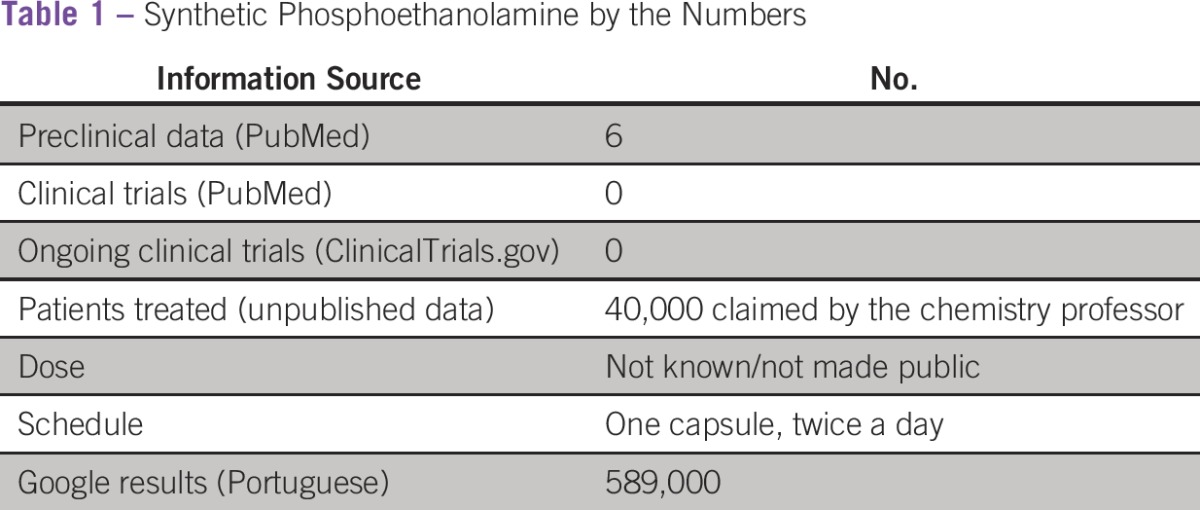
Synthetic Phosphoethanolamine by the Numbers

In October 2015, the case reached one of the Brazilian Federal Court judges, who ruled that patients could continue using the substance and that the university laboratory should not stop making phosphoethanolamine, even though it was not accredited to make compounds for human use. Following this decision, additional lawsuits have been brought forward to guarantee the right to treatment for patients without taking into consideration the lack of evidence and the bypassing of the mandated scientific and regulatory process of drug development. Also in October, a hearing in the National Congress demonstrated the commotion of patients who placed all their hope in the supposed drug and clamored for action. After this meeting, the Ministry of Health created and funded (to the tune of US$2.5 million) a task force to conduct preclinical studies and clinical trials to evaluate phosphoethanolamine as a cancer treatment. The upheaval and pressure from groups of patients and family members have been so forceful and misguided that legislators are currently evaluating the creation of a special track for the regulation of this unproven substance. As of this writing, thousands of lawsuits are making their way through the court systems, and even a fake version of the substance has become available on Internet retailing sites.

Although further study may be appropriate, the uncontrolled distribution and use of the substance without proper evidence of its benefits and safety constitute a hazard to public health in Brazil. We expect that society, lawmakers, and the judicial system will come to understand that and redirect this wanton energy into improving our regulatory system for clinical trials and drug approval, speeding up the availability of truly promising drug candidates and proven medications to our patients.
